# Synthesis and Properties of Flexible Polyurethane Using Ferric Catalyst for Hypopharyngeal Tissue Engineering

**DOI:** 10.1155/2015/798721

**Published:** 2015-07-06

**Authors:** Zhisen Shen, Jian Wang, Dakai Lu, Qun Li, Chongchang Zhou, Yabin Zhu, Xiao Hu

**Affiliations:** ^1^Lihuili Hospital of Ningbo University, Ningbo 315040, China; ^2^The Medical School of Ningbo University, Ningbo 315211, China; ^3^School of Materials Science and Engineering, Nanyang Technological University, Singapore 639798

## Abstract

Biodegradable polyurethane is an ideal candidate material to fabricate tissue engineered hypopharynx from its good mechanical properties and biodegradability. We thus synthesized a hydrophilic polyurethane via reactions among polyethylene glycol (PEG), e-caprolactone (e-CL) and hexamethylene diisocyanate (HDI), and thrihydroxymethyl propane (TMP). The product possessed a fast degradability due to its good wettability and good mechanical parameters with the elongations at break (137 ± 10%) and tensile strength (4.73 ± 0.46 MPa), which will make it a good matrix material for soft tissue like hypopharynx. Its biological properties were evaluated via* in vitro* and* in vivo* tests. The results showed that this hydrophilic polyurethane material can support hypopharyngeal fibroblast growth and owned good degradability and low inflammatory reaction in subcutaneous implantation. It will be proposed as the scaffold for hypopharyngeal tissue engineering research in our future study.

## 1. Introduction

Hypopharyngeal carcinoma is one of highly heterogeneous tumors which develops via chemical carcinogenesis or virus-induced tumorigenesis [1]. Although advancements have been achieved in treating head and neck cancer with adjuvant chemotherapy, radiotherapy, and targeted molecular therapies, surgical intervention is still the main clinical treatment [2]. Inevitably, large defects after surgery will lead to severe voice handicap and swallowing disability. Thus, tissue grafts like a jejunal flap, supraclavicular artery flap, and anterolateral thigh flap are commonly used for surgical repair [3–8]. This will induce tissue defects at other sites and double harms for patients. With the development of tissue engineering, artificial grafts using biomaterials as the matrix have been much studied to repair the tissue defects.

In our previous work, biodegradable poly(ester urethane) (PU, 58213 NAT 022) with good mechanical strength has been used as matrix to test its properties of biocompatibility and degradability. It was found that this PEU material was nontoxic and could well support the growth of skeletal muscle cell and hypopharyngeal fibroblast, which is obtained from animal and human hypopharynx, after it was modified via silk fibroin grafting on the material surface [9, 10]. However, this PEU was a little high modulus and high ultimate stress, which was not favorable for a soft tissue constitution. The degradation seems quite slow too. In order to resolve this issue, we synthesized a new kind of polyurethane with monomers like polyethylene glycol (PEG), L-lactide (L-LA), and hexamethylene diisocyanate (HDI) [11]. This polyurethane possessed a good mechanical properties (very low glass transition temperature, T_g_, −22°C) and high wettability with water uptake of 229.7 ± 18.7%. However, due to the good hydrophilicity, its degradation was too fast comparing with the hypopharynx regeneration; the weight loss in PBS at 37°C was around 45% at day 40.

In this work, polycaprolactone-poly(ethylene glycol)-polycaprolactone (PCL-PEG-PCL) was introduced as the segmented polyester diols to synthesize a degradable polyurethane since the PCL-PEG-PCL segment can enhance the material's hydrolytic process and material's flexibility due to its components of flexible PCL and hydrophilic PEG [12]. PCL-PEG-PCL diols was firstly synthesized with monomers of poly(ethylene glycol) (PEG) and *ε*-caprolactone (*ε*-CL) using iron (III) acetylacetonate as the catalyst [13]. Biodegradable poly(ester urethane) was subsequently obtained via cross-linking of HO-PCL-PEG-PCL-OH, hexamethylene diisocyanate (HDI), and trihydroxymethylpropane (TMP), abbreviated as CPU for simplification. Our house-made mold with microchannels was used to fabricate a scaffold with microchannel pattern at the same time during poly(ester urethane) cross-linking procedure. Physicochemical and morphological characterizations of the scaffold were performed to issue the influence of the ratio of hydrophilic component (PEG) to hydrophobic component (CL) on the CPU's degradability.

Many works verified that 3D pattern on substratum could guide cells to grow orientedly, thus to maintain cells' phenotype and biofunction* in vitro* [14, 15]. As in our previous work, hypopharyngeal skeletal muscle cell was seeded on a microchannel patterned to get cells' alignment [9]. Thinking of construction of tissue engineered hypopharynx in future, we designed a micropatterned, biodegradable, and flexible polyurethane scaffold since both fibroblast and skeletal muscle cells are the main cell types in hypopharyngeal tissue. In order to evaluate the synthesized material's cytocompatibility, human hypopharyngeal fibroblasts (HHF) were seeded on the scaffold. The results showed that this scaffold supported the growth of HHF; cells had good mitochondrion activity and specific protein, vimentin, and secretion. The scaffold was subcutaneously implanted into SD rats to test its biocompatibility and* in vivo* degradation. Surface-induced inflammatory response was assessed by real-time PCR based quantification of proinflammatory cytokine transcripts, namely, TNF-*α* and IL-1*β*. The results demonstrated that the CPU fabricated in this protocol was lower toxic compared with the industry polyurethane. This CPU was believed to be a biodegradable and biocompatible material for soft tissue constitution and regeneration.

## 2. Materials and Methods

### 2.1. Materials

The industry poly(ester urethane) (PU, 58213 NAT 022) was purchased from Estane Co. (China) and was used as reference in this work. *ε*-Caprolactone (CL), poly(ethylene glycol) (PEG, Mn 2000), hexamethylene diisocyanate (HDI), and trihydroxymethyl propane (TMP) were purchased from Sigma-Aldrich and dried by vacuum desiccation for 1 h at room temperature before use. Iron (III) acetylacetonate (Fe(acac)_3_) was purchased from Aladdin Reagent Co., Ltd, China. Dulbecco's modified Eagle's medium (DMEM), fetal bovine serum (FBS), antibiotic antimycotic solution (AAS, 100 U/mL penicillin, 100 U/mL streptomycin), and Trypsin-EDTA solution were purchased from Gibco (Invitrogen Co., New York). Anti-vimentin was supplied by Abcam Ltd. (Hong Kong). FITC-conjugated goat anti-mouse IgG was purchased from Jackson ImmunoResearch Co. Other chemical reagents used in this experiment were from Sinopharm Chemical Reagent. Phosphate buffer saline (PBS, pH = 7.4) used in cell culture was sterilized.

### 2.2. Synthesis and Characterization of PCL-PEG-PCL Macrodiols

**Scheme 1:**
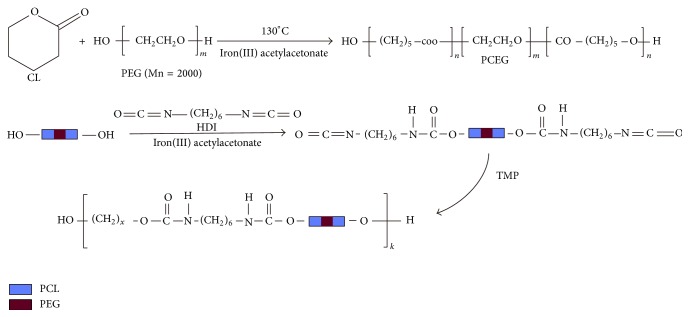
Synthesis of the macrodiols (HO-PCL-PEG-PCL-OH) and CPUs.

PCL-PEG-PCL diols were synthesized via ring-opening polymerization with Fe(acac)_3_ as the catalyst (Scheme 1). In a typical process, monomers CL, PEG (with variable molar ratios, Table 1), and Fe(acac)_3_ (1 wt% of total monomers) were reacted for 24 h at 140°C under argon protection. The product (PCL-PEG-PCL diols) was thus obtained. For the sake of simplification, PCL-PEG-PCL diols with various CL/PEG ratios were abbreviated as PCEG1–3 (Table 1). It was dissolved in dichloromethane and precipitated in methanol and subsequently dried in vacuum oven for 24 h. Their number-average molecular weights (Mn) were determined by gel permeation chromatography (GPC, Polymer Laboratories PL-GPC 50 plus, England) and listed in Table 1. The reaction chemistry of PCL-PEG-PCL macrodiols was confirmed via nuclear magnetic resonance (^1^H NMR) and Fourier transform infrared (FTIR) measurement. ^1^H NMR spectra of the macrodiols were recorded on a 400 MHz Bruker spectrometer using CD_3_Cl as the solvent. The FTIR spectra were obtained on a FTIR spectrometer (Digilab FTS 3100, USA).

### 2.3. Synthesis and Characterization of Cross-Linked Poly(Ester Urethane) (CPU)

CPUs were synthesized from PCL-PEG-PCL diols and HDI using TMP as a chain extender by a two-step method. The molar ratio of PCL-PEG-PCL : HDI : TMP is 1 : 2 : 1. In a typical process, PCL-PEG-PCL diols were firstly distilled in toluene in a three-necked flask and then dissolved in anhydrous 1,4-dioxane (DO) to get PCL-PEG-PCL solution (6% mg/mL). HDI and Fe(acac)_3_ (1 wt% of PCL-PEG-PCL and HDI) were added into the solution to let the reaction occur for 2 h at 70°C. After cooling to room temperature, TMP dissolved in 1,4-dioxane (2.68 g/mL) was added dropwise and reacted for 7 h. The whole synthesizing process was protected by argon. The cross-linking yields of CPU were >95%. CPU membrane (~1.0 mm Thickness) was prepared via casting the product solution onto a plane surface and dried in air overnight for the following characterization. For the sake of simplification, the cross-linked products with the corresponding macrodiols reagents were abbreviated as CPU1–3 (Table 2).

The thermograms of CPU were conducted on a differential scanning calorimetry instrument (DSC, Pyris Diamond, USA) at a heating rate of 10°C/min with nitrogen flow. The temperature interval of the DSC analysis was 0.2°C. The first heating ranged from −90 to 100°C at a speed of 10°C/min with 2 min station to clear the thermal history and then cooled down to −90°C at a speed of 10°C/min. The second heating started from −90 to 100°C at 10°C/min. Values of glass transition temperature (T_g_) and melting point (*T*_*m*_) were taken from the second heating round.

The droplet dynamic contact angles of CPUs were tested on Data physics OCA20 (Germany) at ambient humidity and temperature. Drop of deionized water was 1.0 *μ*L in volume. Each sample was examined three times at different locations. Before measurements, samples were dried at 30°C under vacuum for 24 h.

Degradation test was performed for CPU3 in PBS (pH 7.4) containing with penicillin- (100 U/mL) streptomycin (100 *μ*g/mL). In brief, weighed samples (*W*_0_) of CPU3 were incubated in PBS containing 100 U/mL Penicillin-streptomycin that was changed twice per week. pH value was stable during the whole test. The industry PU was used as the reference. Samples were dried for 12 h in vacuum oven at 60°C and weighed as *W*_1_. The weight loss was calculated as (*W*_0_ − *W*_1_)/*W*_0_ × 100%.

CPU3 were chosen to test the tensile strength properties. They were cut into Dumbbell-shaped (0.2-0.3 mm × 1 mm, *n* = 3) and tested on a linear tensile tester (Instron 3366, USA) at a linearly deformed rate of 10 mm/min at room temperature. Three repeats were performed for each sample.

### 2.4. Patterned Scaffold Preparation

Based on our previous work, a soft polydimethylsiloxane (PDMS) mould was fabricated from a silica wafer patterned with unidirectional microchannels of 200 *μ*m in width and separated by walls with 30 *μ*m wide and 30 *μ*m high. The patterned CPU films (thickness of 150 *μ*m) were prepared by casting CPU/1,4-dioxane solution onto this PDMS mould followed by solvent evaporation for 12 h at 37°C, yielding a transparent CPU membrane with parallel microchannels of 200 *μ*m width and 30 *μ*m depth. The membrane was then immersed in alcohol solution (95 v%) for 3 h, rinsed with large amounts of water, and dried in air.

### 2.5. Cytocompatibility Tests

#### 2.5.1. Cell Culture

Human fibroblasts were obtained from hypopharyngeal tissues, sacrificed by someone with laryngeal or hypopharyngeal carcinoma at the Otolaryngology Department of Lihuili Hospital of Medical School, Ningbo University (Ningbo, China). Only specimens which were determined to be free of cancer by the pathologist were used. Collection of biopsy samples was approved by the Ethics Boards of Ningbo University with the consent of patients. The biopsy specimens stored in PBS (pH = 7.4, ice-cool) supplemented with 100 U/mL penicillin-streptomycin upon collection were immediately transported to our laboratory for further processing. The tissue samples were rinsed with PBS, and the fat and tendons were removed with ophthalmic scissors. The clear muscle tissues were then minced into pieces of approximately 1 × 1 × 1 mm and transferred into culture flask (Corning, USA), supplemented with 2 mL of DMEM containing 15% FBS and 100 U/mL penicillin-streptomycin. The medium was subsequently renewed every three days. Cells migrated from tissues to attach onto the flask after 7 days. Cells from 2nd to 5th passages were used for cytocompatibility test.

#### 2.5.2. Cell Activity Assay

Number of the cells (Figure 3) on scaffolds was assayed using the Cell Counting Kit-8 (CCK-8) at day 2, day 5, and day 10, respectively. 10 *μ*L of the cck-8 solution was added to each culture well (in 96-well plate) and incubated for 4 h at 37°C in dark. The absorbance was measured at 490 nm using an ELISA reader (MaxM5, Spectra). Data were compared between cells grown on PU, CPU3, and TCPS. Triplicates of each sample were averaged.

#### 2.5.3. Immunofluorescence Staining

Cells cultured on scaffolds, CPU3 for 10 days, were fixed in 4 wt% paraformaldehyde (Sigma, USA) for 30 min and rinsed three times with PBS for 10 min each time. The samples were immersed in 0.2% Triton X-100 for 10 min, rinsed three times for 10 min each time with PBS, and then blocked in 4% goat serum for 1 h in order to minimize nonspecific binding. The entire process was carried out at room temperature. The blocking solution was drained from the samples (without washing) and incubated overnight in anti-vimentin mouse monoclonal antibody (1 : 200 dilution in PBS) at 4°C. After rinsing with PBS three times for 10 min each time, the samples were subsequently incubated in anti-mouse IgG secondary antibody conjugated with FITC (1 : 100 dilution in PBS) for 1 h in the dark. For nuclei observation, the samples were dipped in 4,6-diamidino-2-phenylindole dihydrochloride (DAPI) solution (Sigma, 3 *μ*g/mL in PBS) and immediately rinsed with PBS. In the stained image, the vimentin displayed green florescence while the nuclei displayed blue florescence.

#### 2.5.4. Subcutaneous Implantation

Female SD rats (3 months old, 250–300 g) were assigned into 2 groups, 8 rats for each group. Each rat was implanted subcutaneously by two sterilized PU and CPU3 membranes (the same size as 1 well of 96-well culture plate) after the rat was given an analgesic (ketoprofen 1.5 mg) and anesthetized with 5% chloral hydrate (intraperitoneal injection, 6 mg/kg). After 7 and 30 days, the rats were sacrificed. The samples were carefully explanted with some surrounding tissue and HE staining was performed.

The animals used in this study were treated in accordance with the ethical committee of Ningbo University and NIH's Principles of Laboratory Animal Care.

#### 2.5.5. Hematoxylin and Eosin (HE) Staining

Samples were fixed in 4% paraformaldehyde at 4°C. Fixed samples were rinsed with water, dehydrated through a graded series of alcohol, made transparent with dimethyl benzene, wax-dipped, embedded in paraffin, sectioned at 4 *μ*m, dewaxed, hydrated, and stained with H&E dye. The stained samples were observed under light microscopy (Olympus CX40, Japan). Images were captured by digital camera (PL-B623CU, Pixelink, Canada).

#### 2.5.6. Real-Time PCR Analysis

The inflammatory response to the implants was evaluated by quantifying the local gene expression of the proinflammatory cytokines, namely, TNF-*α* and IL-1*β*, after 1 month implantation. Tissues from the explanted polymer implants were carefully scrapped using a sterile surgical blade in a sterile Petri plate. Total RNA was extracted from tissues using Trizol reagent (Ambion, Carlsbad, CA) according to the manufacturer's instructions. The GoScript Reverse Transcription System (Promega, Madison, WI) was used to generate combinational cDNA. To evaluate TNF-*α* and IL-1*β* levels, real-time quantitative reverse transcriptase-polymerase chain reaction (qRT-PCR) was achieved using the GoTaq qPCR Master Mix (Promega) on an Mx3005P Real-Time PCR System (Stratagene, La Jolla, CA). The sequences of the PCR primers for glyceraldehyde-3-phosphate dehydrogenase (GAPDH), TNF-*α*, and IL-1*β* were listed in Table 3. The conditions of thermal cycling were as follows: 10 minutes at 95°C for a hot start; then 45 cycles at 94°C for 15 seconds, 55°C for 30 seconds, and 72°C for 30 seconds. The cycle threshold (Ct) values were recorded for TNF-*α*, IL-1*β*, and the housekeeping gene GAPDH. The expression levels of TNF-*α* and IL-1*β* were calculated using the ΔCt method with GAPDH as the control to normalize the data. Lower ΔCt values indicate higher expression. All results were expressed as the mean ± standard deviation of 3 independent experiments.

### 2.6. Statistical Analysis

Data are expressed as mean ± standard deviation (SD). Statistical comparisons were made by analysis of variance (ANOVA). *t*-test was used for evaluations of differences between groups. *P* values less than 0.05 were considered to be significant.

## 3. Results and Discussion

### 3.1. Synthesis of PCL-PEG-PCL Macrodiols

**Figure 1:**
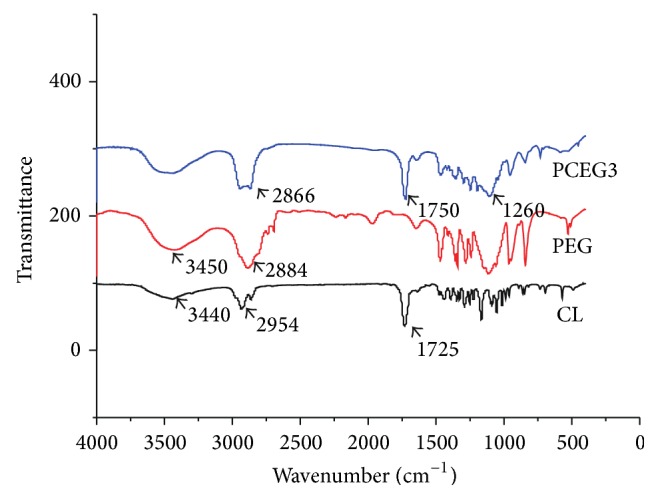
FTIR spectra of CL, PEG, and PCEG3.

PCL-PEG-PCL diols (PCEG) were synthesized from monomer *ε*-caprolactone (CL) and oligomer PEG with molecular weight of 2000 Dalton using low toxic iron compound as the catalyst (Scheme 1). The effect of various ratios between CL and PEG on the product's molecular weight was explored (Table 1). The average molecular weights of PCEG obtained by GPC are in the range of 7015–17422 Da, increasing with the increase of CL amount. The low polydispersity (~1.8–2.0) of the PCEG indicated a narrow distribution of the molecular weights as a consequence of good control on the polymerizing process. All PCEGs were successfully synthesized as demonstrated by FTIR spectroscopy (Figure 1), which exhibited spectrum of CL, PEG, and PCEG3. Curve PCEG showed the summary peaks of PEG and monomer CL with a specific peak at 1725 cm^−1^ which can be attributed to ester stretching absorption of CL and PCEG, and a wide peak at 3450 cm^−1^ which can be attributed to hydroxyl groups in the end group of PEG and PCEG molecule (very weak peak in CL).

**Figure 2:**
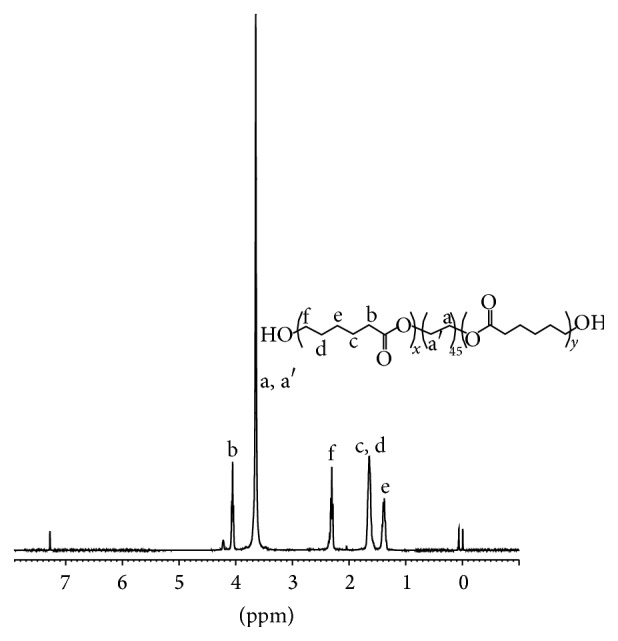
^1^H NMR spectrum of prepolymer PCEG3.

The fact that the terminal hydroxyl groups of PEG can be readily activated by Fe(acac)_3_ to further function as coinitiator for the ring opening polymerization of CL towards forming triblock macrodiols (PCL-PEG-PCL, PCEG). The quite low molecular weights can improve the processability and solubility of the polymers; meanwhile, high molecular weight polyurethanes may possess high stiffness. The molecular structure of three PCL-PEG-PCL macrodiols, that is, PCEG1, PCEG2, and PCEG3, was also confirmed by ^1^H NMR measurement (Figure 2). Both protons of methyl CH_2_ at 1.57 ppm (c, d) and of CH with its tertiary carbon attached to carbonyl groups at 4.05 ppm (b) from CL monomer showed the polycaprolactone (PCL) block, while protons of CH_2_CH_2_ segment attached to two oxygen atoms at 3.64 ppm (a, a′) were determined to originate from PEG moiety. These results, ^1^H NMR together with FTIR characteristics, confirmed the occurrence of polymerization between PEG and CL to yield PCEG macrodiols, HO-PCL-PEG-PCL-OH, PCEG in short.

### 3.2. Properties of CPU

The cross-linking of PCEG and HDI was conducted with TMP as the extender, to yield the cross-linked polyurethane (CPU) (Scheme 1). CPU membrane was fabricated via casting the reaction solution onto a PDMS mold. The membrane was then washed in alcohol, rinsed in water, and dried in oven overnight.

The properties of CPU were evaluated via thermal behaviors, mechanical property, wettability, and degradation test. From DSC measurements, the glass transition temperature (T_g_) and melting point (*T*_*m*_) of CPU3 appeared at −51 and 38°C (Table 2), which implied that this CPU was an elastomer and will meet the mechanical requirements for artificial materials to be used as bio-matrix for soft tissues. However, CPU1 and CPU2 seem a bit hard and frangible at room temperature, which might be caused by low CL content in molecular structure. Therefore, we will mainly focus on the CPU3 for the following experiments.

**Figure 3:**
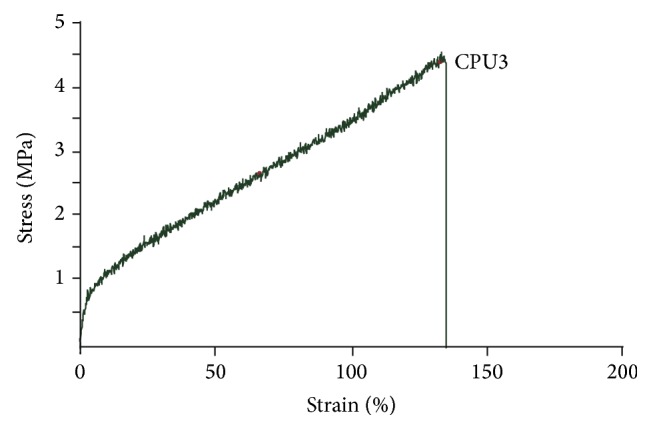
The stress-strain curve of CPU3 membrane.

The mechanical properties of CPU3 were displayed on stress strength-strain curve (Figure 3). The maximum strength and ultimate strain were recorded as 4.73 ± 0.46 MPa and 1.37 ± 0.10 mm/mm, which was stronger than natural materials like collagen (0.31 Mpa) [16] or chitosan (0.55 Mpa) [17] and biodegradable polymers like polyvinyl pyrrolidone (0.35 Mpa) [18] or aloe vera (1.5 Mpa) [19], both of which were often used as biomedical application.

The scaffold's mechanical properties were affected by the PEG component under the same quenching process. CPU3 demonstrated good tensile stress and strain (4.73 ± 0.46 MPa and 1.37 ± 0.10 mm/mm). The ultimate strain was better than the previous polyurethane which was cross-linked from PEG, HDI, and L-lactide [11]. CL in components of the polymer greatly promoted the material's flexibility. We considered that CPU3 had higher cross-linking degree than CPU1 and CPU2, resulting in the better tensile strength and higher strain. Because the samples were manufactured from the random cross-linking of three different components, that is, PCEG, HDI, and TMP, higher cross-linking degree led to the lower regularity or crystallization degree in molecular structure. The maximum strength and ultimate strain of nature larynx had been tested to be 9.65 ± 0.24 MPa and 1.42 ± 1.31%, respectively [11]. The nature larynx seems stronger than the synthesized CPU3. We supposed the similar or a little softener property of Hypopharynx than larynx from eye-viewing. However, we are short of the detail data due to shortage of the tissue at present.

**Figure 4:**
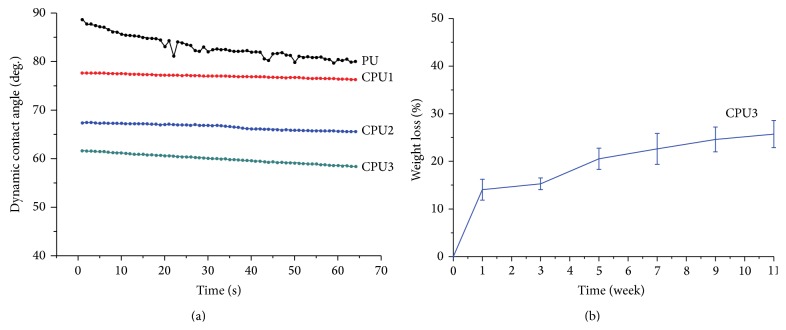
Dynamic contact angle (a) and weight loss (b) of CPU as a function of time. The specimens were immersed in sterilized PBS (pH 7.4) at 37°C and kept in a sealed container during weight loss measurement.

An ideal material for tissue engineering application should possess good hydrophilicity and biocompatibility. Thus, a hydrophilic PEG component was introduced. The surface hydrophilicity of CPU was evaluated via dynamic contact angle measurements (Figure 4(a)). It showed that the dynamic contact angle (CA) of CPU decreased dramatically with increasing the contents of PEG, that is, CPU1–CPU3. Particularly, CA of CPU3 reached to 55 degree in 64 seconds. The wettability of CPU1 or CPU2 was poorer than that of CPU3, but still better than that of the industry PU, which changed slowly from 80 to 89 degree in 64 seconds. Improvement in wettability was expected to promote cytocompatibility to hypopharynx cells; on the other hand, it will enhance material's hydrolysis rate. As the degradation tests during 11-week span (Figure 4(b)), CPU3 lost 14.1, 15.3, 20.5, 22.6, 24.6, and 25.7% of their initial weight after 1, 3, 5, 7, 9, and 11 weeks, respectively. It is reasonable because segmented polyester based polyurethane with highly units of PEG led to increasing degradation rate (increasing weight loss) due to its high hydrophilicity. Thus, CPU3 sheet displayed faster degradation rate. Improved hydrophilicity of these polymers possesses the relatively fast degradation behaviors [20, 21].

**Scheme 2:**
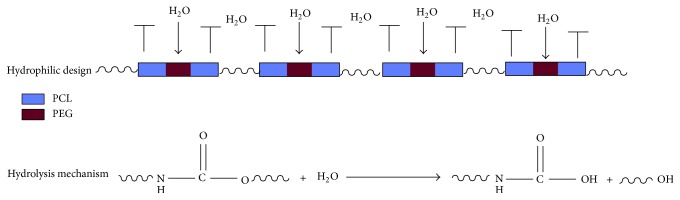
Mechanism of CPU hydrolysis in PBS.

The hydrolysis mechanism was conjectured as Scheme 2 described. The hydrophilic unit, PEG, can remarkably attract H_2_O molecules to attack urethane bond, leading to material's hydrolysis. Within degradation process of CPU, tissue can replace it for rebuilding function and avoiding second operation. The surface wettability is an important index for hydrophila which is often determined by measuring the dynamic contact angle of films [22].

### 3.3. Cytocompatibility to Primary Hypopharyngeal Fibroblasts

**Figure 5:**
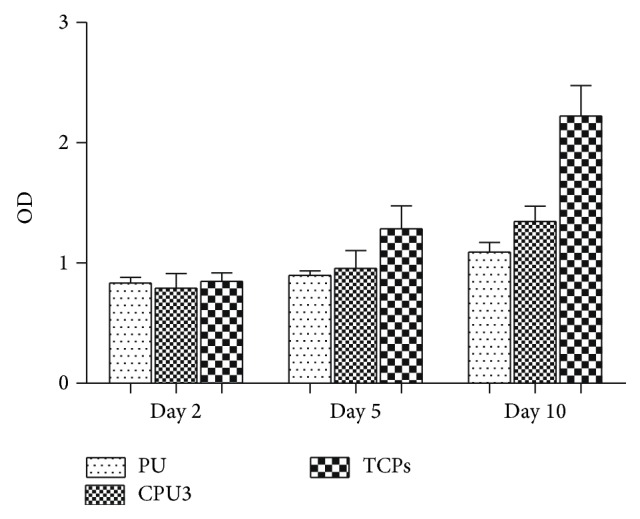
Hypopharyngeal fibroblasts growth on CPU3 membrane. Cell counting assay: relative absorbance at 490 nm.

**Figure 6:**
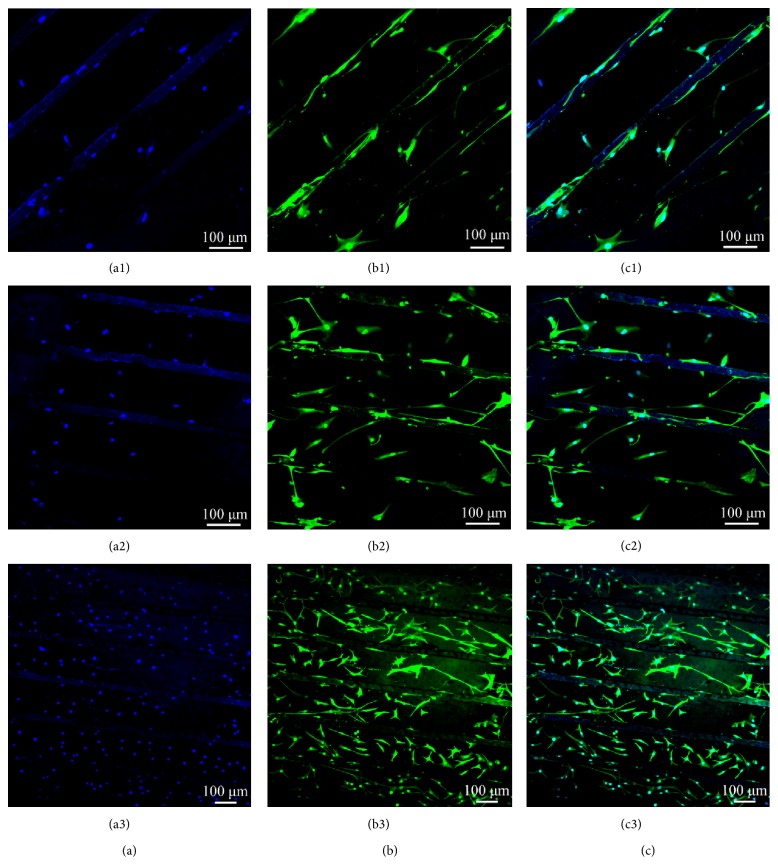
Immunofluorescence staining: cell nuclei were stained with DAPI (a). Cell cytoplasm was stained with antivimentin (b). (c) was composited by (a) and (b). Cells were seeded at the density of 5 × 10^4^ cells mL^−1^ and cultured for 2, 5, and 10 days (1, 2, and 3), respectively.

Because of main cell components of hypopharyngeal tissue, primary hypopharyngeal fibroblasts were seeded on CPU3 membrane with the industry PU as control. The membrane was patterned with microchannels in order to guide cells alignment.  Figure 5 shows the growth tendency of fibroblasts. On CPU3 membrane, cells spread and attach well after being cultured for 2 days. The fibroblast's growth tendency during 10-day span displayed a step-up increase of cell number (Figure 6). Though the absolute number of fibroblast on CPU3 is lower than that on TCPS at the culture stage, it is tending to be faster than the control PU. In addition, human primary fibroblasts were evaluated using immunoflorescence analysis. Figure 6 showed the long spindle morphologies of fibroblast cells on the surface after they were cultured for 2 days. Using antivimentin as the primary antibody, cells were immunohistochemically stained and exhibited positive phenotype to confirm their human fibroblast origin. After being cultured on the microchannels for 10 days, cells displayed spread and adhered very well onto CPU matrix. Moreover, it exhibited alignment along the channel direction. These results indicated that fibroblasts could survive and grow well on the scaffold using the present culturing methodologies. We will conduct systemically study on the regeneration of hypopharyngeal tissue with biological constitution and function in the next work.

### 3.4. *In Vivo* Biocompatibility of Scaffolds

**Figure 7:**
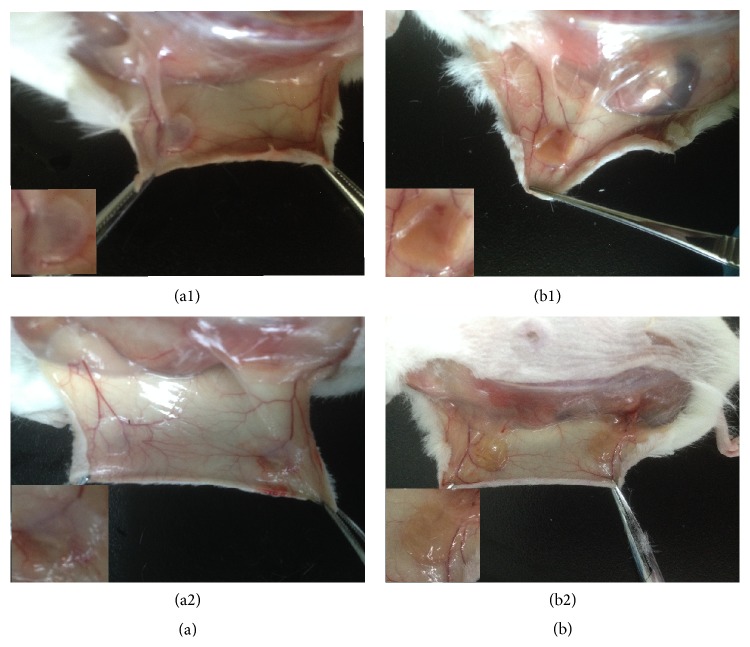
The photographs of implanted PU (a) and CPU3 (b) after they were implanted in SD rat subcutaneously for 7 d (1) and 30 d (2). The inserted images showed the tissue encapsulated PU and CPU3 membranes.

CPU3 scaffolds were implanted subcutaneously into SD rats to detect their biocompatibility* in vivo* and degradability within tissue regeneration using control PU as the comparison (Figure 7). The operated wound of both CPU3 and PU recovered completely at day 7. We can find that there was no redness and swelling that occurred during the first week after the operation in CPU3 group (b1), whereas tissue adhesions happened in the control (PU, a1). After 30 d, the control PU scaffold (a2) was still a little filthy in background and inflammatory exudate was apparent. In comparison, CPU3 (b2) was more homogeneous and did not cause any hemolysis, pyrogen, or hyperergia with the surrounding tissue throughout the research, accompanied with some angiogenesis. These results implied that hydrophilic CPU synthesized using ferric catalyst was more biocompatible to animal tissue than the hydrophobic PU scaffold using tin compound catalyst [12].

**Figure 8:**
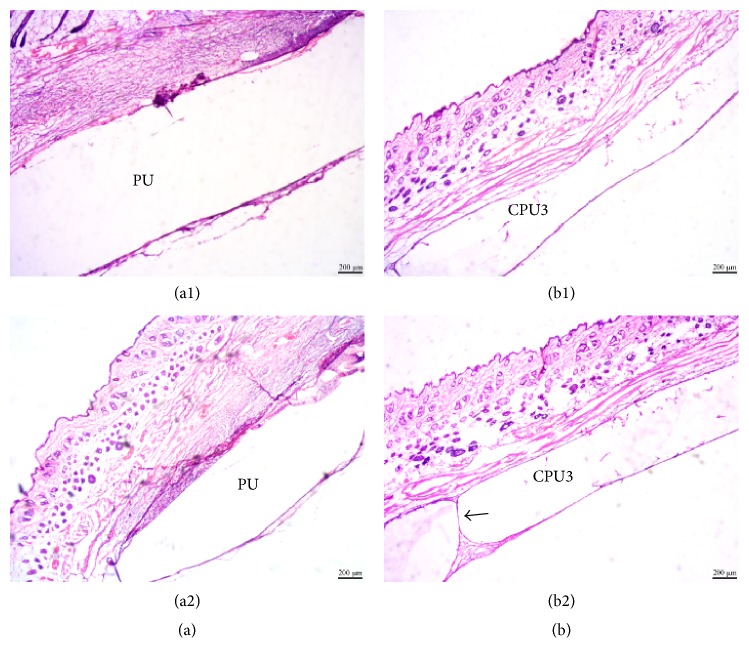
Hematoxylin and eosin (H&E) staining. The control PU (a) and CPU3 (b) were implanted for 7 d (1) and 30 d (2). Scale bar 200 *μ*m.

In order to investigate scaffolds' biocompatibility, degradability, and tissue infiltration, scaffolds with tissue were histologically analyzed by H&E staining. At day 7, tissues and scaffold (the middle white region) were demarcated clearly for both CPU3 and PU (Figure 8). Results displayed that there are more eosinophilic granulocyte obviously in PU group (a1) than CPU3 (b1) at 7 day. As the polyester type material degradation, granulation tissue infiltrated into CPU3 bulk (b2) and connected the two sides of tissue with the time passing by, which might be attributed to scaffold's high hydrophilicity, faster biodegradability, and better cytocompatibility. However, tissue hardly penetrated into the hydrophobic, slow biodegradable PU group (a2). Thus, hydrophilic, biodegradable segmented polyurethane scaffolds can promote tissue infiltration.

### 3.5. Real-Time PCR Analysis of Proinflammatory Cytokine Gene Expression Level

**Figure 9:**
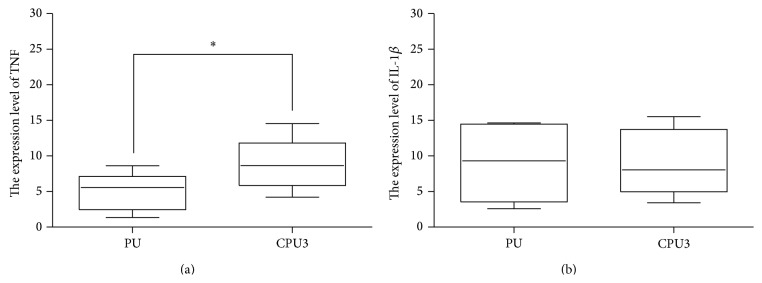
The expression levels of TNF-*α* and IL-1*β* in tissue response for PU and CPU3 implanted subcutaneously for 30 days, as determined by real-time PCR analysis (mean ± SD, ^*∗*^ *P* < 0.05).

In order to further guarantee the safety of artificial polyurethane scaffolds, the inflammatory response to CPU3 and control PU were evaluated by quantifying the expression levels of the cytokines interleukin-1*β* (IL-1*β*) and tumor necrosis factor (TNF-*α*) after they were implanted in SD rat. The sequences of the PCR primers for GAPDH, TNF-*α*, and IL-1*β* were listed in Table 2. The expression levels were calculated using the Δ cycle threshold (ΔCt) method. *P* < 0.05 was considered to be significance.  Figure 9 demonstrated that the level of IL-1*β* in two groups seemed to be of no significant difference, but TNF-*α* was downregulated in CPU3 group compared with PU (*P* < 0.05), which is consistent with the results of HE stained. Although foreign body reaction is poorly understood, the proinflammatory signals play a crucial role in the whole process included in cascade of activities: stimulation of T-cells, recruitment of leukocytes, motivation of neutrophil oxidative metabolism, cell proliferation, and fibrosis [23, 24]. The lower level of foreign body reaction in CPU3 group may be contributed by the high hydrophilic property of materials, which was expected to enhance cell-scaffold interaction. Hydrophilic surfaces were shown to significantly, in our previous reports, strengthen the biocompatibility compared with hydrophobic surfaces. Another reason may be that CPU3 is made by ferric catalyst which is lower toxic than heavy metal tin in activating inflammatory response. These studies presented the effectiveness of the hydrophilic CPU3 surface in decreasing the expression levels of proinflammatory cytokines by immune system. As to the micromolecule hydrolysis products of biomaterials whether completely eliminated from the body, further study like isotope labelling to elements was necessary [25].

## 4. Conclusions

In this study, using iron compound Fe(acac)_3_ as the catalyst, a series of macrodiols (PCL-PEG-PCL) with different molecular weights was synthesized. The reaction was testified by FTIR spectroscopy, ^1^H NMR spectra and GPC, and so forth. After these macrodiols reacted with HDI and TMP, the high-hydrophilic cross-linked polyurethane was produced under different reactant contents and molar ratios. The properties like thermodynamics, mechanical behavior, surface hydrophilicity, and degradability of CPU1, CPU2, and CPU3 were tested and discussed. CPU3, whose mechanical property is similar to the hypopharyngeal tissue, was determined to be the optimal substrate for further* in vitro* and* in vivo* research.

Its cytocompatibility to primary hypopharyngeal fibroblasts was quantitatively tested and qualitatively evaluated by CCK8 and immunohistochemistry. The results verified that hydrophilic CPU materials can support hypopharyngeal fibroblast cells growth with good biocompatibility.* In vivo* investigation demonstrated that CPU material possessed good degradability and low inflammatory reaction. Within hydrophilic scaffold of CPU3 hydrolysing, tissues regeneration through the scaffold took place after it was implanted subcutaneously in SD rats for 30 days without inflammatory cells. The results of real-time PCR analysis of proinflammatory cytokine gene expression (IL-1*β* and TNF-alfa) were studied to further prove the biosecurity of this material. We thus concluded the potential of this hydrophilic CPU scaffolds with aligned microchannels for future applications in hypopharyngeal tissue engineering.

## Figures and Tables

**Table 1:** Macrodiols PCEG composition, molecular weight, and molecular weight distribution.

Macrodiols	CL/PEG in feeding (mol)	Mn (Da)	Mw (Da)	Mw/Mn
PCEG 1	70/1	17422	35275	2.0247
PCEG 2	35/1	11924	24788	2.0788
PCEG 3	17.5/1	7015	12597	1.7957

**Table 2:** The composition and appearance of CPUs.

Cross-linked polyurethane	Macrodiols	Diisocyanate	Chain extender	State at room temperature	T_g_ (°C)	*T*_*m*_ (°C)
CPU 1	PCEG 1	HDI	TMP	Hard	−45	46
CPU 2	PCEG 2	HDI	TMP	Frangibility	−48	44
CPU 3	PCEG 3	HDI	TMP	Elasticity	−51	38

**Table 3:** Primer sequences used for the real-time PCR analysis.

Gene target	Primer sequence	Orientation
TNF-*α*	5′-AAATGGGCTCCCTCTCATCAGTTC-3′	Forward
	5′-TCTGCTTGGTGGTTTGCTACGAC-3′	Reverse

IL-1*β*	5′-CACCTCTCAAGCAGAGCACAG-3′	Forward
	5′-GGGTTCCATGGTGAAGTCAAC-3′	Reverse

GAPDH	5′-GTATTGGGCGCCTGGTCACC-3′	Forward
	5′-CGCTCCTGGAAGATGGTGATGG-3′	Reverse
